# Dishabituation of the BOLD response to speech sounds

**DOI:** 10.1186/1744-9081-1-4

**Published:** 2005-04-22

**Authors:** Jason D Zevin, Bruce D McCandliss

**Affiliations:** 1 Sackler Institute for Developmental Psychobiology, Weill-Cornell Medical College, 1300 York Ave, Box 140, New York, NY USA

## Abstract

**Background:**

Neural systems show habituation responses at multiple levels, including relatively abstract language categories. Dishabituation – responses to non-habituated stimuli – can provide a window into the structure of these categories, without requiring an overt task.

**Methods:**

We used an event-related fMRI design with short interval habituation trials, in which trains of stimuli were presented passively during 1.5 second intervals of relative silence between clustered scans. Trains of four identical stimuli (standard trials) and trains of three identical stimuli followed by a stimulus from a different phonetic category (deviant trials) were presented. This paradigm allowed us to measure and compare the time course of overall responses to speech, and responses to phonetic change.

**Results:**

Comparisons between responses to speech and silence revealed strong responses throughout the extent of superior temporal gyrus (STG) bilaterally. Comparisons between deviant and standard trials revealed dishabituation responses in a restricted region of left posterior STG, near the border with supramarginal gyrus (SMG). Novelty responses to deviant trials were also observed in right frontal regions and hippocampus.

**Conclusion:**

A passive, dishabituation paradigm provides results similar to studies requiring overt responses. This paradigm can readily be extended for the study of pre-attentive processing of speech in populations such as children and second-language learners whose overt behavior is often difficult to interpret because of ancillary task demands.

## Background

Habituation effects have been observed in a wide range of neural systems from simple sensory responses [1], to higher-order neural representations such as motion-sensitive populations in Area MT [2], and regions responding to written and spoken language [3]. We can take advantage of neural habituation to study the preattentive categorization of stimuli. By presenting a single speech stimulus repeatedly, we can observe habituation to that sound, then by comparing this condition to one in which a "deviant" stimulus occurs after a series of repeated "standards," we can also determine which brain regions are sensitive to the change between the two stimuli (see review in [4]).

Critically, habituation phenomena can be studied with passive paradigms, which have tremendous advantages in the study of speech perception, particularly for the study of populations in the process of acquiring language, or adult populations differing in their early language experience. Any overt task involves a range of decision processes that can act to obscure the processes underlying speech perception under more natural conditions. This is a particular problem for studying speech developmentally, because attention and decision processes develop very slowly [5,6] which may cause us to underestimate children's ability to perceive phonetic contrasts. Furthermore, adults are often quite good at discriminating sounds in laboratory tasks that they do not perceive phonetically [7]. Even after extensive training on perception and production, it can be difficult to establish whether second language learners are using the same underlying mechanisms as monolinguals, even when many surface aspects of behavior are similar between the two groups (see, for example, [8]). Neuroimaging can help establish how stimuli are discriminated, for example by showing differential activity in regions specifically implicated in phonetic processing. The goal of the current study is to develop a short interval habituation trial paradigm optimized for event-related fMRI designs that builds on the strengths of currently available methods, and can be applied to a range of populations of interest.

### Developing an auditory habituation paradigm for fMRI

The mismatch negativity (MMN) response, as observed in EEG (and its equivalent mismatch field response in MEG) is a form of neural dishabituation that has been used as an index of the categorization of speech sounds [4]. For example, Naatanen et al. [9] presented stimuli from the partially overlapping vowel systems of Finnish and Estonian to native speakers of each language. They found smaller MMN responses for a deviant stimulus that was not a native-language vowel, even though it was acoustically more different from the standard than a non-native stimulus. Another major advantage of this technique is that the MMN can be observed in the absence of any attention-demanding task. Typically, subjects in such studies are reading or watching a film, but characteristic mismatch responses have also been observed in sleeping infants and comatose patients (see Cheour et al. [10], for review).

There has been increasing interest in combining the advantages of the MMN paradigm with the higher spatial resolution available using fMRI. For example, in a study by Fiez and colleagues [11], spoken words were presented repeatedly and responses were observed to both repeated words and occasional deviants. A comparison between these conditions revealed responses in temporal regions involved in speech and auditory processing, as well as frontal and parietal regions implicated in attentional function.

A number of technical challenges complicate this approach. A very real challenge to these studies is the noise created by the MR scanner itself, particularly at high field strengths, and when using scanning sequences optimized to provide higher signal to noise ratios [12]. Thus, there is a trade-off between the strength and resolution of the signal and the ability to present acoustic stimuli in relative quiet.

Another trade-off exists between optimization of the stimulus presentation parameters for interpretation of the dependent measure (the blood oxygenation level dependent, or BOLD response) and paradigm optimization for the observation of change responses. The BOLD response evolves very slowly, making it difficult to observe "baseline" responses to rapidly repeated stimuli typical of MMN designs. When stimuli are presented at a constant rate, the BOLD response from any stimulus cannot easily be deconvolved from responses to previous stimuli. On the other hand, the temporal jittering of stimuli critical to fast event-related designs [13] is undesirable because mismatch responses are reduced when stimuli are not presented at a constant rate [14]. As we show below, directly comparing time course information from standard and deviant stimuli can provide novel insights into the role of particular brain regions in the perception of phonetic change.

In the current study we address a number of these methodological challenges by presenting short interval habituation trials composed of trains of four stimuli. On "standard" (STD) trials, these consisted of four repetitions of the same speech sound, whereas on "deviant" (DEV) trials, three repetitions of one sound were followed by a different, dishabituating sound (see also [15,16]). We used a clustered data acquisition protocol in order to present stimuli in silence, and combined a relatively short repeat time (i.e., whole brain volumes obtained every 3 seconds) with a long inter-trial interval to maximize our ability to derive time series from individual subjects' data.

The results demonstrate the feasibility of the methodology, and reveal responses to passively presented phonetic stimuli. In particular, activity in the left posterior superior temporal gyrus related to phonetic change is robust at the single-subject level and may serve as a signature for automatic categorization of speech sounds.

## Results

Two separate analyses were undertaken. In the first, we compared responses from trials on which speech was presented (collapsing across standard and deviant trials) to silence, in order to observe the BOLD signal for passive perception of speech. In the second analysis, we directly compared the deviant and standard trials, in order to study responses to phonetic change. We discuss each analysis in turn.

### Responses to speech versus silent baseline (SPCH > SIL)

**Figure 1:**
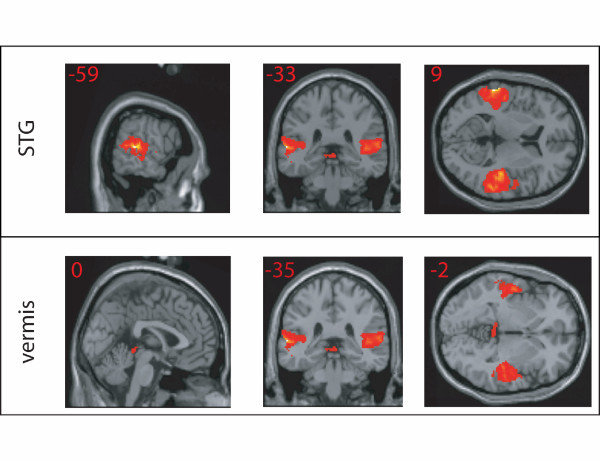
**SPCH > SIL activations**. Areas of greater activation for speech (SPCH) than silence (SIL); voxels thresholded at p < .005 uncorrected. Note the lack of activity in Heschl's gyrus (top), likely the result of stimulation by the acoustic noise of the scanner.

As shown in Figure 1, in comparisons between speech and silence, the STG is active bilaterally from its most posterior extent up to but not including the temporal pole (activity in the anterior portions of the temporal lobe is difficult to observe in fMRI, [17]). This is consistent with the well-established role these regions play in auditory processing, in particular for speech [11,18-20]. This pattern of response was observed consistently across all subjects.

As shown in Table 1, we also observed cerebellar responses to speech stimuli bilaterally in the superior portion of the vermis. Numerous neuropsychology studies have linked damage in this area to disorders of speech production [21]. Interestingly, activity in the vermis has also been observed in auditory perception experiments in which stimuli are presented rhythmically, for example trains of tones or frequency modulated sweeps [22] or clicks [23] and pairs of words [24]. Thus, it is possible that the cerebellar responses in this task reflect rhythmic properties of the stimulus presentation paradigm.

**Table 1:** Regions of significant activation in the SPCH > SIL contrast

Talairach coordinates	Area	Z(voxel)	cluster size
-59, -33, 9	Left STG	5.50	1754
-51, -8, -3		4.45	
-51, -21, 3		4.25	
			
44, -25, 10	right STG	5.29	2512
53, -21, 3		5.14	
50, -29, 5		5.07	
			
0, -35, -2	vermis	3.15	53
-10, -33, 0,		2.79	

Note: SPCH > SIL = increased response to speech relative to silence; STG = superior temporal gyrus. Cluster size is based on a voxel-wise threshold of p < 0.005 uncorrected.

### Responses to phonetic change (DEV > STD)

Responses to phonetic change were observed in a broad network of regions, including a portion of left STG known to play a role in phonological processing (see Table 2). Activity was also observed in regions implicated in novelty responses, and right hemisphere regions potentially involved in processing of extra-phonetic aspects of the stimuli.

**Table 2:** Regions of significant activation for the DEV > STD contrast

Talairach coordinates	Area	Z(voxel)	cluster size
Left			

-51, -36, 24	STG/SMG	3.84	122
-40, -34, 22	border	3.41	
-42, -33, 29		3.31	
			
-20, 57, 21	MFG, BA10	4.49	57
-20, 52, 27		2.71	
			
-28, 27, 45	MFG	3.75	45
-30, 18, 47		2.91	
-20, 18, 49		3.36	
			
-24, -41, 43	Parietal	4.13	30
-24, -38, 50		2.95	
			
-40, -72, 42	Precuneus (BA19)	3.44	36
-40, -64, 36		2.89	
-40, -74, 33		2.78	
			

Right			

32, -9, -15	Hippocampus	4.65	40
			
4, -23, 14	Thalamus	3.40	64
4, -13, 10	(medial dorsal)	2.73	
			
53, -17, 14	Postcentral (BA3)	3.95	52
46, -11, 17		3.50	
			
55, -2, 6	R Precentral, BA6	4.52	35
			
28, -7, 17	Putamen	3.12	27
			
36, 0, 42	MFG (BA6)	3.43	27
44, 0, 44		2.80	

Note: DEV > STD = increased response to deviant trials relative to standard trials; STG = superior temporal gyrus; SMG = supramarginal gyrus; MFG = middle frontal gyrus; BA = Brodmann's Area. Cluster size is based on a voxel-wise threshold of p < 0.005 uncorrected.

### Dishabituation of the BOLD response in posterior left STG/SMG border

**Figure 2:**
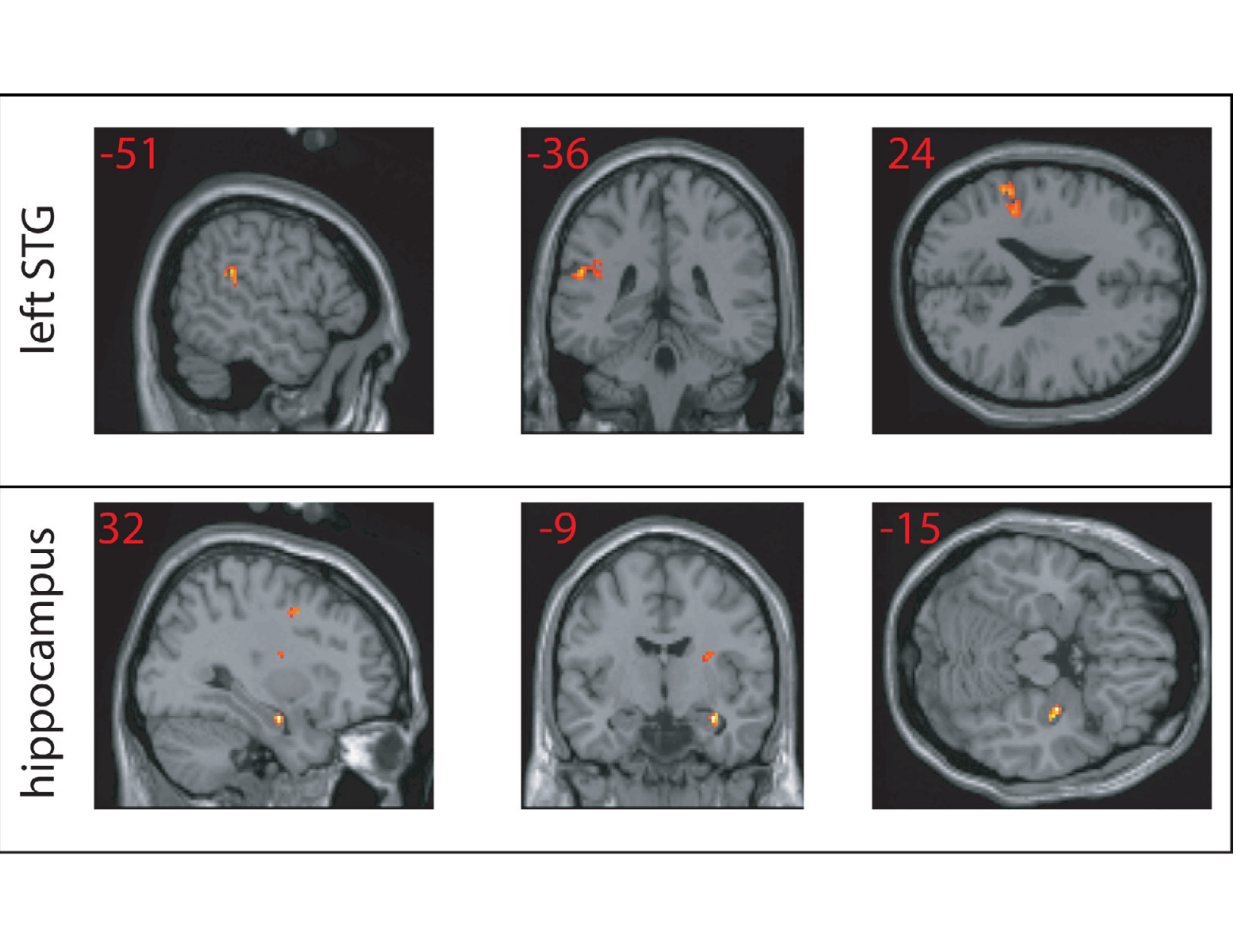
**DEV > STD activations**. Areas of greater activation for deviant (DEV) than standard (STD); voxels thresholded at p < .005 uncorrected. The perisylvian region (top) is actually located along the border of superior temporal and supramarginal gyri.

A restricted region of left posterior STG responds preferentially to trials containing a phonetic change (DEV) in comparison with trials containing no phonetic change (STD), as shown in Figure 2. This region is along the border with SMG and has been reported in a number of studies involving active phonetic change judgments [25,26]. This region also shows a marginally significant response to the speech relative to silence contrast, voxelwise t(7) = 2.63, p < .05.

**Figure 3:**
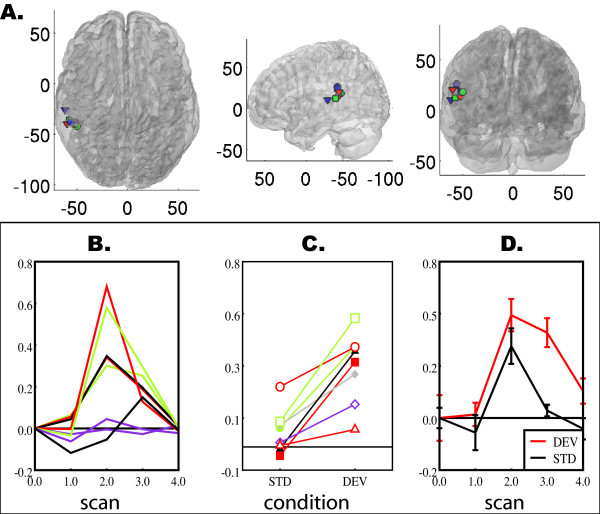
**Peak activations and time series from individual subjects for the DEV > STD contrast**. A. Peak activations for the DEV > STD contrast in each subject nearest to the group mean plotted on a translucent rendering of grey matter. B. Time series for responses to all speech stimuli in each individual's peak., C. Mean percent signal change at scans 2 and 3 (approximately 2–7 seconds after the fourth syllable in each train) for STD and DEV trials. D. Mean time series for DEV and STD for all subjects.

We observed a high degree of consistency across subjects in the pattern of activity in this region. The peak activations nearest this region are plotted on a translucent rendering of the MNI template brain in Figure 3A. Every individual had a peak BOLD response in the DEV > STD contrast close to the peak in the random effects analysis (mean euclidean distance = 8.58, range = 4.47 – 19.70, SD = 5.12). Time series for an 8 mm sphere around the mean peak (mean number of active voxels in sphere = 106.75, SD = 16.67) are plotted for each subject in Figure 3B. Note that for most subjects, this region shows subthreshold responses to speech overall, whereas in all subjects, responses to DEV trials are greater than responses to STD trials (Figure 3C). This can be seen in the mean time series (Figure 3D) for all 8 subjects. Critically, the pattern observed here is consistent with a dishabituation response: The region typically responds to speech, yet its response is attenuated when the same stimulus is presented repeatedly; when a novel stimulus is presented, a heightened response is observed.

**Figure 4:**
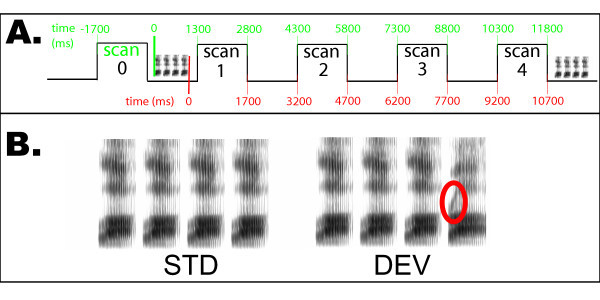
**Short interval habituation trials paradigm**. Schematic diagram of the stimulus presentation paradigm. A. Timing of scans relative to stimulus presentation, taking as the stimulus onset either the onset of speech sounds (in red) or the fourth stimulus which distinguishes deviant from standard trials (in green). B. Spectrograms of standard (STD) and deviant (DEV) stimulus trains. The primary phonetic difference between the /la/ and  is the onset frequency of the third formant, circled in red.

We also observed timecourse differences between responses to speech and responses to phonetic change. Given the timing of scans relative to auditory stimulation (Figure 4), and the fact that the peak of the hemodynamic response occurs between 4 and 6 seconds [27], we would expect responses to the onset of speech stimuli to occur at around scan 2 and responses to the deviant stimulus to occur around scan 3. In fact, there is a difference in when the peak response to speech (median = scan 2) occurs and the peak difference between deviant and standard stimuli (median = scan 3), which is reliable according to a Wilcoxon signed-ranks test, W = 21, n_*s*/*r *_= 6, p < .05.

### Novelty responses

The hippocampus is known to be involved in novelty detection, in keeping with its role in the encoding of novel episodic memories [28]. In the current experiment, an anterior region of right hippocampus responded preferentially to deviant trials, consistent with this role. Two recent studies of neural responses to novel stimuli have found similar activations in hippocampus: Kiehl et al. [29] presented novel (low-probability) natural sounds in the context of an auditory target detection task with sine wave tones as the baseline stimuli. In addition to bilateral hippocampal activations, they observed novelty responses in regions of left superior temporal and superior frontal gyrus, right postcentral gyrus, and the medial dorsal nucleus of the thalamus similar to the current study. In a similar task using visual stimuli, Yamaguchi et al. [30] found hippocampal responses, as well as left superior frontal, right middle frontal and left parietal activations similar to the current study.

### Right hemisphere responses

A number of right hemisphere regions also responded more strongly to deviant trials than to standards. Although our paradigm did not require any motor response, and did not result in any primary motor or somatosensory activity, the pattern of BOLD signal in the right precentral gyrus, postcentral gyrus and putamen, was similar to that observed in studies of disparate motor and proprioceptive responses [31-35]. One feature that the current experiment shares with other tasks in which these regions have been activated is the rhythmic presentation of acoustic stimuli. For example, in the Joliot et al. study [31], subjects were required to tap their fingers in time with a tone. In the current study, no motor response was required, but trains of stimuli were presented in a regular rhythm (see Figure 4). It is unclear, however, why these regions would respond preferentially to deviant trials whereas the vermis responds to rhythmic aspects of both STD and DEV conditions.

One peak in right precentral gyrus (BA6, Tc = 55, -2, 6) appears from inspection of individual subjects to reflect activity in two different regions: a region of precentral gyrus that has been found in many of the same conditions as the post-central region discussed above, and a portion of right anterior STG. This may result from the fact that, using natural stimuli for which both steady-state and transitional portions differ between the standard and deviant stimuli, there are some unavoidable extraphonetic differences between the stimuli that may activate right superior temporal regions involved in the processing of tone timbre and amplitude [19,22,36].

## Discussion

A central motivation for this study was to map out the spatial topography and timecourse of BOLD responses in regions generally sensitive to speech stimulation, and regions sensitive to changes in the speech signal. We also sought to test whether such responses might be collected under passive conditions similar to those in electrophysiology research using mismatch negativity designs, which have proven useful in developmental and cross linguistic studies in which explicit discrimination and labelling may introduce confounds. The use of short interval habituation trials affords the possibility of directly contrasting the topography and time course of responses to speech stimuli and phonetic habituation.

### Characterizing neural responses to speech stimuli and phonetic change

This study demonstrates that under passive presentation conditions, BOLD responses to short trains of syllables inserted within the 1.5 seconds of relative silence between volume acquisitions are reliably obtained in a broad network of superior temporal gyrus regions. This includes both early auditory areas involved in the processing of spectrally complex sounds [37,38] and regions that are potentially specific to speech processing [19,39].

We also found that passive presentation of a short interval of habituation, (i.e. four syllables in rapid succession), establishes sufficient context to generate dishabituation effects related to the change of single phoneme. A restricted region of posterior, left STG, along the border with supramarginal gyrus responded contrastively to information in the fourth syllable, such that a novel phonetic onset produced a greater response relative to the habituated one. This suggests a role for this region in phonetic processing, consistent with several lines of converging evidence. First, this region is active in explicit phonetic discrimination [25] and comparisons of passive listening to speech with other stimuli [40]. Furthermore, in a study of native Japanese speakers learning English, activity in this region is correlated with accuracy in discriminating English speech sounds [26]. These findings suggest that responses in such regions may be relatively specific to phonetic change.

Although regions of the superior temporal gyrus have been implicated in mismatch negativity studies using simple sine-wave tones as stimuli, the regions observed in the current study are distinctly posterior and superior to the putative location of the mismatch negativity responses for those relatively simple stimuli [41,42]. In a direct comparison of passive responses to stimulus change for speech stimuli and tones, Celsis et al. [20] found speech-specific responses in a region of left supramarginal gyrus contiguous with the extent of the activity observed along the STG/SMG border in the current study. Furthermore, a similar region is specifically activated by sine-wave stimuli when they are perceived as speech compared to the same stimuli when they are perceived as oscillating tones [43]. Finally, the posterior portion of left STG is involved in reading and seems in particular to be critical to aspects of decoding that require mapping of visual letters onto speech sounds [44-46]. Taken together, the data suggest a critical role for this region in the passive perception and categorization of speech sounds.

### Advantages and disadvantages of using short interval habituation trials in event related designs

Short interval habituation trials provide a fast, passive paradigm, with which it is possible to observe robust data in small numbers of subjects. One critical advantage of short interval habituation trials design is that it allows us to examine relationships between responses to syllables and effects of phonetic habituation. Because the entire time course of activation is collected after each trial, it is possible to examine the response to speech stimulation in general and habituation specifically. For example, in Figure 3D, the BOLD signal for standard trials is consistent with general sensitivity to speech, but the response to deviant trials has a time course consistent with dishabituation to the fourth stimulus in a second-long train. The ability to extract a complete time series from each trial is an improvement over the silent paradigms with much longer repeat times used in earlier studies [20,41]. In those paradigms, images are acquired every 10–12 seconds, so that the BOLD response to scanner noise returns to baseline between scans. This means that data are collected at a single discrete time point for each trial. While this makes it possible to isolate regions that respond more strongly to a run of stimuli containing deviants than a run containing only standards, it only provides time course information when stimuli are presented at multiple delays relative to the data acquisition.

The main disadvantage of the current technique relative to slower designs is the lack of sensitivity to early auditory processing. For example, activity was not observed in left primary auditory cortex for either of the contrasts examined (speech > silence, deviant > standard). This may be due to the loud acoustic noise generated by the flipping of the gradients in the in-out spiral sequence which is likely to activate neurons in this region. If this region is activated by recurring scanner noise, the BOLD response may become saturated during the experiment, limiting the ability to observe speech-related responses in the current paradigm. Thus, there is a design tradeoff between the efficiency with which change-related responses to speech and early auditory processes can be observed.

It may be possible to combine the current approach with a long inter-trial interval in order to observe the contribution of early auditory processing to speech perception. Belin et al. [37] developed a presentation paradigm in which individual stimuli are presented at different times relative to the onset of scanning on each trial. In this way, it is possible to reconstruct time course information by combining responses from multiple trials. Because the trains of stimuli used in the current design are quite brief (just over one second), it would be possible to present these trains in a similar manner, allowing us to observe the contribution of early auditory areas to phonetic change perception. Possible advantages of this technique may be outweighed by practical concerns. By using a short repeat time to sample time course information at 5 intervals for each short interval habituation trial, we collected full data sets for a two-condition contrast in a relatively short time (approximately 26 minutes of scanning). An experiment that took five times this long to collect data for a single pair of stimuli would not be practical in many cases. In particular, if one wanted to compare responses to native language stimuli with responses to non-native stimuli [25,26] it would require multiple scanning sessions.

Thus, while it has disadvantages for observing low-level auditory activity, the short interval habituation trial paradigm has a number of features that make it particularly applicable to studies of different populations of interest. The lack of any explicit task allows for investigation of responses to speech stimuli under processing conditions similar to well established procedures in mismatch negativity research. Furthermore, passive presentation of auditory stimuli while subjects are engaged in an unrelated visual activity (i.e. watching a video) reduces the influence of attentional and executive factors. This eschews difficulties with overt tasks where such factors may lead to an underestimation of children's performance, or to strategic attention to extra-phonetic cues that may lead to an overestimation of performance by adult second-language learners [7]. The granularity of the time series data is also critical in the analysis of data from developmental and cross-linguistic studies: In cases where no dishabituation effect is observed, establishing that some response to speech sounds is observed makes it less likely that the null result is a type I error. Finally, this study demonstrates robust data at the level of individual subjects, providing a strong basis for the study of individual variability in populations of interest.

## Conclusion

Using short interval habituation trials, we were able to isolate specific regions of superior temporal gyrus that are sensitive to changes in phonetic information in the absence of any explicit instructions or task in adult native English-speaking subjects. Many theoretical questions central to the investigation of speech processing revolve around comparisons of listeners of different ages and from different language backgrounds. The current approach can be extended to yield insights into the development and plasticity of the basic mechanisms that subserve phonetic perception.

## Methods

### Subjects

Eight right-handed adult native English speakers (ages 23–38, mean = 26.8, SD = 4.6, 2 females) participated in the experiment. Subjects were paid for their participation.

### Stimuli

Natural speech sounds used in the experiments consist of recordings of an adult male native English speaker (JDZ) saying the syllables  and /la/ with a similar pitch and intonation pattern. Stimuli were digitized in 16-bit mono at 22050 Hz and cropped to 250 ms in duration using Praat [47] by deleting individual glottal pulses from the steady-state portion of the vowel. Recordings were made in a soundproof booth at the Speech and Hearing Research Center of the City University of New York Graduate Center.

### Stimulus presentation

On each trial, a train of four stimuli was presented. On "standard" trials the same stimulus was repeated four times; on "deviant" trials the final stimulus differed from the first three. Silent trials were also included to allow a baseline for comparison. The stimuli had a duration of 250 ms, and were presented with an inter-stimulus interval of 50 ms, so that the duration of an entire stimulus train was 1.15s. This allowed us to leave 175 ms of silence between both the onset and offset of the auditory stimuli and the spiral data acquisition time to prevent auditory masking. Stimuli were presented at approximately 70 dB. The IFIS system, combined with EPrime (both from Psychology Software Tools) software was used to synchronize stimulus presentation with the scan sequence.

The experiment consisted of 12 pseudorandomized functional runs of 9 trials each, with an ITI of 12s. In each block, equal numbers of standard, deviant and silent trials were run. In the first six blocks,  served as the standard stimulus and /la/ was the deviant, and in the last six blocks this was reversed. Over the course of a full session, this provided 36 trials for each stimulus type (standard, deviant, silence). Each run began and ended with an extra 12 s silent trial in order to account for heterogeneity in the magnetic field at the beginning of scanning runs and to provide a full acquisition period for the final stimulus train in a block.

Because of the high level of acoustic noise generated by the spiral sequence (~120 dB), subjects were supplied with 30 db attenuating foam earplugs. In preliminary tests, this did not interfere with hearing the stimuli, but provided protection from the noise of the scanner. In addition to the earplugs, subjects were fitted with a large pair of padded piezo-electric headphones which provided additional protections from sound. We also used Tempurpedic pillows to fill in parts of the headcoil. This served two purposes: First, it aided in noise abatement. We have found that the headcoil itself acts to amplify acoustic noise by acting like a resonating body. By preventing the headphones from coming into direct contact with vibrating parts of the headcoil and filling in empty space directly around the subject's head this effect is reduced. The foam also helped subjects remain still. Finally, in order to make the experiment less tedious for subjects, nature films or cartoons were shown on the video monitor during stimulus presentation. Films were shown continuously throughout the session, providing a pattern of visual stimulation highly unlikely to be correlated with any experimental procedure.

### Data acquisition

After an initial three-plane localizer and a whole-head coronal localizer, a Fast Spin Echo sequence was taken in an axial-oblique plane prescribed to correct for head position in 3 dimensions (obviating the need for manual AC-PC alignment later in processing) TR = 3325 ms, TE = 68 ms, flip = 90°, FOV = 22, 5 mm slice thickness, 0 mm gap, matrix = 256 × 192, 20 slices, positioned to cover language and auditory processing regions. High-resolution T1-weighted images for normalization were taken using a 3D gradient echo SPGR sequence, axial plane, TR = 25 ms, TE = 5 ms, flip = 20°, FOV = 24 cm, 1.5 mm slice thickness, 0 mm gap, matrix = 256 × 256 × 160.

Functional images were taken using the spiral in-out sequence developed by Glover and colleagues, and the same spatial prescription as the FSE, TR = 3000 ms, TE = 40 ms, matrix = 64 × 64. using a clustered acquisition sequence with a 3s TR and 1.5s TA. By using a clustered acquisition protocol, we are able to present stimuli in relative quiet, i.e., during 1.5s gaps during which no acoustic noise from the flipping of the gradients is present. This scanning sequence has been shown to have a very high signal to noise ratio [12]. Each functional run lasted 132s during which 44 volumes were collected.

### Data analysis

We analyzed fMRI data using SPM2 in three major stages: pre-processing to retrieve the functional data and map all subjects into a common space; statistical parametric mapping to find regions with interesting patterns of activity and follow-up analyses using percent signal change estimates from regions of interest identified in the parametric maps.

### Pre-processing

The first four volumes in each scanning session were deleted to allow the magnetic field to reach steady state. Slice-timing correction was then applied to account for the fact that slices are acquired in fixed order during a 1.5s TA for each 3s TR. Next, image realignment was applied to all functional images, generating a set of realignment parameters for each run and a mean functional image which was used to coregister functional scans to the FSE in-plane anatomical images. The FSE was then coregistered to the SPGR, and these parameters were applied to the functional scans. The SPGR was then normalized to MNI space resulting in oversampled voxels of 2 mm^3^. These parameters were applied to the realigned, smoothed functional images, and the normalized data smoothed using a FWHM kernel of 6 mm.

### Statistical Parametric Mapping

Statistical models were constructed by convolving the onsets of each trial type with a standard hemodynamic response function, including realignment parameters as covariates. These were used to generate first-level contrast images for each subject for two contrasts: 1) SPCH > SIL, showing the pattern of positive correlation with the presence of any speech stimulus relative to silent baseline and 2) DEV > STD showing the pattern of greater responses to deviant trials relative to standard trials. These contrast images served as the basis for random effects analyses. Results reported as significant exceed a voxel-wise threshold of p < .005 and a spatial extent threshold of 25 contiguous voxels. This provides a conservative estimate of statistical significance [48].

### Time series analyses

In order to examine the time series in posterior left STG for standard and deviant stimuli, a functional region of interest (ROI) was defined based on the mean image from all subjects in the DEV > STD contrast. For each subject, an 8 mm sphere was drawn around this ROI and eigenvectors extracted using the VOI toolkit for SPM2. This provided a representative response for the region over time, which was then averaged for each stimulus type to generate a mean time series.

## Authors' contributions

The experiment was conceived, developed and reported collaboratively by both authors. JDZ was primarily responsible for data collection and analysis.
